# Cell Senescence in Heterotopic Ossification

**DOI:** 10.3390/biom14040485

**Published:** 2024-04-16

**Authors:** Robert J. Pignolo, Frederick S. Kaplan, Haitao Wang

**Affiliations:** 1 Department of Medicine, Section of Geriatric Medicine & Gerontology, Mayo Clinic, Rochester, MN 55905, USA; 2 Divisions of Endocrinology and Hospital Internal Medicine, Department of Physiology and Biomedical Engineering, Mayo Clinic, Rochester, MN 55905, USA; 3 Robert and Arlene Kogod Center on Aging, Mayo Clinic, Rochester, MN 55905, USA; wang.haitao@mayo.edu; 4 Department of Orthopaedic Surgery, The Perelman School of Medicine of the University of Pennsylvania, Philadelphia, PA 19104, USA; frederick.kaplan@pennmedicine.upenn.edu; 5 Department of Medicine, The Perelman School of Medicine of the University of Pennsylvania, Philadelphia, PA 19104, USA; 6 The Center for Research in FOP and Related Disorders, The Perelman School of Medicine of the University of Pennsylvania, Philadelphia, PA 19104, USA; 7 Department of Physiology and Biomedical Engineering, Mayo Clinic, Rochester, MN 55905, USA

**Keywords:** cellular senescence, fibrodysplasia ossificans progressiva, heterotopic ossification, senotherapeutics

## Abstract

The formation of bone outside the normal skeleton, or heterotopic ossification (HO), occurs through genetic and acquired mechanisms. Fibrodysplasia ossificans progressiva (FOP), the most devastating genetic condition of HO, is due to mutations in the *ACVR1/ALK2* gene and is relentlessly progressive. Acquired HO is mostly precipitated by injury or orthopedic surgical procedures but can also be associated with certain conditions related to aging. Cellular senescence is a hallmark of aging and thought to be a tumor-suppressive mechanism with characteristic features such as irreversible growth arrest, apoptosis resistance, and an inflammatory senescence-associated secretory phenotype (SASP). Here, we review possible roles for cellular senescence in HO and how targeting senescent cells may provide new therapeutic approaches to both FOP and acquired forms of HO.

## 1. Fibrodysplasia Ossificans Progressiva and Acquired Forms of Heterotopic Ossification

Fibrodysplasia ossificans progressiva (FOP; OMIM#135100) is the most severely disabling genetic disorder of heterotopic ossification (HO). FOP is characterized by congenital malformations of the great toes, progressive endochondral HO and accelerated features of aging [1,2,3,4]. The worldwide prevalence of FOP is 0.5 to 1.36 per million individuals [5,6,7]. There is no ethnic, racial, gender, or geographic predilection to FOP. Patients with FOP undergo acute inflammatory episodes in the context of a chronic inflammatory state [8,9]. Beginning in the first decade of life, episodic bouts of painful soft tissue swellings (exacerbations or flare-ups) occur, which are often precipitated by soft tissue injury, intramuscular injections, viral infection, muscular over-stretching, falls or muscle overuse and fatigue [1,3,10]. These flare-ups transform skeletal muscles, tendons, ligaments, fascia, and aponeuroses into heterotopic bone, making movement impossible. The natural course of FOP is almost always relentlessly progressive, and most patients are near completely immobilized by the third decade of life.

Classic FOP is caused by an activating mutation (617G > A; R206H) in *ACVR1/ALK2* [11]. Patients with atypical forms of FOP have been described [12] and these variants also have heterozygous *ACVR1* missense mutations in conserved amino acids but may present with milder or more severe phenotypes. One case of resilience to FOP in a patient harboring the classic *ACVR1* 617G > A; R206H mutation has been described [13]. The diagnosis of FOP is performed by clinical evaluation and, where available, confirmatory genetic testing. Most cases of FOP are sporadic (non-inherited mutations), but a few cases show germline transmission and complete penetrance in an autosomal dominant pattern [3].

Premature aging in FOP could be primarily related to an *ACVR1/ALK2* mutation (see below), secondarily due to disuse and immobilization, or elements of both [4]. Features of accelerated aging in FOP that could secondarily be due to immobilization from HO and joint ankyloses include diminished lung volumes, osteopenia/osteoporosis, fractures, and muscle atrophy. As examples, the unloading of normotopic bone related to HO bridging predisposes to bone loss, and progressive chest wall rigidity lessens elastic recoil and the force generation of the respiratory musculature predisposing to diminished vital capacity. Sarcopenia in FOP may be due to both disuse atrophy (i.e., joint ankyloses preventing movement) and overactive activin A signaling associated with greater muscle catabolism and the inhibition of muscle cell differentiation. 

In contrast to FOP, acquired or nonhereditary heterotopic ossification (NHHO) tends to be limited rather than progressive and usually arises in the setting of connective tissue or neurological trauma, certain arthropathies, or following an injury that is often sustained in the context of age-related pathology. CNS, musculoskeletal, cutaneous, and vascular injury predisposes an individual to HO, and NHHO occurs as a clinically severe complication in as many as 19% of all individuals following major hip surgery, and in as many as 11% of those following traumatic brain injury [14,15]. HO may occur in the ligaments of individuals with seronegative spondyloarthropathies or diffuse idiopathic skeletal hyperostosis. Individuals who present with NHHO, especially when trauma-induced or associated with orthopedic procedures, tend to have recurrence with subsequent injuries or procedures, and due to largely unknown predisposing factors.

Age-onset conditions may also be associated with NHHO, including those with vascular pathophysiology and degenerative joint disease requiring hip arthroplasty. HO can be found in end-stage calcific valvular disease in up to about 15% of older patients [16]. Age-related HO can occur in a variety of other conditions associated with immobilization (e.g., neuromuscular disorders) or with predisposing clinical risk factors common in the geriatric population, such as pressure sores, urinary tract infection, or trauma. Pulmonary ossification is much less common but can occur with the chronic medical problems often found in older individuals, including left ventricular failure and secondary hyperparathyroidism [17]. Like FOP, NHHO is characterized by a critical threshold of injury that induces inflammation and hypermetabolism, which precipitously dysregulates normal tissue repair [18,19]. This critical threshold is likely much higher in NHHO compared to FOP.

## 2. Lesion Formation in Fibrodysplasia Ossificans Progressiva and Non-Hereditary or Acquired Forms of Heterotopic Ossification

Both in FOP and in acquired forms of HO, lesion progression, at least in terms of histological stages, appears to be very similar. In the early lesion, soft tissue destruction ensues, especially in skeletal muscle, and the necrosis of myocytes in a hypoxic microenvironment is accompanied by a massive inflammatory infiltrate composed of lymphocytes, macrophages, and mast cells [20,21,22,23,24,25,26,27,28,29]. An intense fibroproliferation (i.e., fibrosis) and neovascularization precede chondrogenesis and, finally, endochondral ossification. In both FOP and NHHO, lesions tend to be mosaic, with multiple histological stages represented in the same tissue section. In preclinical models and in patient biopsy tissue, senescent cells can be detected in early lesions showing muscle necrosis, inflammatory infiltration and fibroproliferation [30].

## 3. Possible Roles of Cellular Senescence in Heterotopic Ossification

Cellular senescence is a hallmark of aging and thought to be a tumor-suppression mechanism [31]. Characteristic features of cellular senescence include irreversible growth arrest, apoptosis resistance, and the senescence-associated secretory phenotype (SASP), which describes an inflammatory secretome with local and systemic deleterious effects. Mitochondrial dysfunction and alterations in DNA and chromatin organization are additional prominent features. Senescent cell burden in animal models of aging has been associated with geriatric syndromes, chronic diseases, multimorbidity, and premature aging phenotypes. In preclinical models, the genetic or pharmacologic clearance of senescent cells facilitates the delay or amelioration of a wide range of aging-related pathology and functional declines [31].

Cellular senescence may be induced by multiple deleterious factors such as reactive metabolites, DNA damage, oncogenes, reactive metabolites, mitogens, proteotoxic stress, inflammation, as well as damage-associated molecular patterns (DAMPs) and/or microbial pathogen-associated molecular patterns (PAMPs), and a DNA damage response is required for induction [31]. Depending on the inducer(s) and cell type(s), cellular senescence is established by downstream pathways that ultimately activate p16INK4a/Rb and/or p53/p21. In the context of FOP lesion formation leading to HO, several of these inducers appear relevant, including inflammation, DAMPS/PAMPS, mitogenic signaling, and the reactive metabolites related to tissue injury.

Although HO is a prominent feature of FOP, we have also described early-life changes in individuals who have FOP that recapitulate the features of accelerated aging [31]. Plausible mechanisms for the generation of a premature aging phenotype include the accumulation of senescence cells after injury and overactive activin A signaling [30]. Injury-induced senescence has been reported in muscle [32,33] and muscle injury is a major cause of flare-ups in FOP [2,3]. Increased bone morphogenetic protein (BMP) pathway signaling, especially by activin A, has also been implicated in osteoarthritis, sarcopenia, neurodegeneration and other clinical features shared between aging and FOP [31]. In addition, activin A is a factor described as part of the SASP [34,35] and the mutated *ACVR1/ALK2* BMP type I receptor in FOP aberrantly responds to activin A [36]. Thus, it is possible that injury-induced senescence (i.e., the increased production of activin A) may initiate FOP exacerbations and propagate BMP signaling via *ACVR1* activating mutations that subsequently promote age-related changes in various tissues [31].

In FOP, disease progression can occur without patient-reported signs or symptoms and without obvious clinical manifestations of flare-ups. This is reported to occur in almost 50 percent of patients [10]. There may be several explanations for this, including sub-acute lesion formation, aborted lesion formation (i.e., the lack of progression to ossification), the continued progression of fibroproliferative or cartilaginous stages to ossification after the resolution of a clinical flare-up, or accelerated osteoarthritis [10,37]. Cellular senescence may be implicated in FOP progression, even in the absence of episodic flare-ups, since their accumulation in tissues would conceivably proceed in subclinical soft-tissue injury, with disuse atrophy and with normal aging. Independently of HO formation, senescence cells may exert their effects locally and systemically through SASP factors that are known to contribute to FOP progression in preclinical models and in patients (e.g., activin A, inflammatory cytokines).

## 4. Cellular Senescence and Urist’s Fundamental Concepts of HO Formation

**Figure 1:**
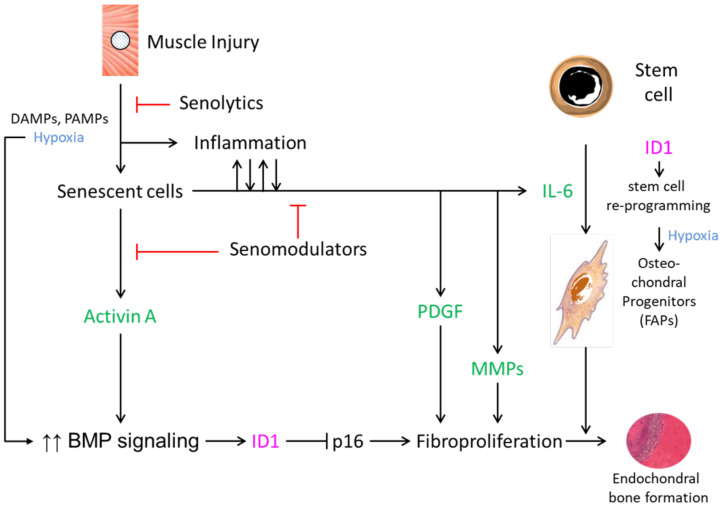
Potential mechanisms by which cellular senescence contributes to heterotopic ossification in FOP (based on [30]). Key SASP factors are shown in green. Activation (→), inhibition (┴), upregulation (↑↑).

The requisite events required for endochondral heterotopic bone formation in any genetic or acquired context, as first described by Urist, include the following: an inflammatory trigger, an inductive signal, a receptive progenitor cell, and a conducive microenvironment [38]. Flare-ups in FOP are precipitated by trauma, muscle overuse, and viral infection. A working model, in which senescence makes a principal contribution to the pathogenesis of HO in FOP (Figure 1), postulates that under these clinical circumstances, exacerbations of FOP are precipitated by inflammatory triggers. Tissue damage is mediated by endogenous DAMPs or PAMPs, especially in muscle undergoing hypoxic changes [29,39]. As the result of DAMPs or PAMPs and potentially reactive metabolites created by muscle injury, senescent cells accumulate and, through their SASP, facilitate the inductive pathways that enhance BMP signaling and the reprogramming of tissue-resident stem cells [30] (Figure 1).

Senescence at the cellular level can be caused by a variety of insults, including the response to tissue damage characterized by an irreversible cell cycle arrest and by the SASP [40,41]. The SASP is associated with the production of proinflammatory cytokines, matrix metalloproteinases (MMPs), growth factors, and two key factors that can increase BMP signaling and stem cell reprogramming—activin A and IL-6, respectively. In FOP cells, activin A is a known potent inducer of BMP signaling [36,42,43,44,45,46]. In addition to its direct secretion by senescent cells, elevated activin A production may also be enhanced by activated MMPs in lesional tissue, which permit the migration of inflammatory cells from leaky and hypoxic capillaries, the degradation of the extracellular matrix, and the subsequent release of activin A and other osteogenic BMP/TGF-β family ligands from matrix-bound heparan sulfate proteoglycans [47,48]. 

Beyond the permissive role of IL-6 in cellular reprogramming, FOP cells also demonstrate an increased efficiency of induced pluripotent stem cell (iPSC) generation [49]. For example, in normal cells, iPSC generation is greatly enhanced by transducing mutant ACVR1 or SMAD1 or by adding BMP4 during early reprogramming. In fact, inflammatory responses in iPSC-derived macrophages are extended by ACVR1(R206H) signaling [50]. Furthermore, the activation of NF-kappa B/MAPK signaling appears to be associated with ACVR1-promoted inflammatory responses in HO formation in humans [8]. 

A major barrier to cellular reprogramming is p16/INK4A-mediated cell senescence. Inhibitors of DNA binding and cell differentiation (ID) genes, the transcriptional targets of BMP-SMAD signaling, are critical for iPSC generation and can suppress p16 through the BMP-SMAD-ID signaling axis which is overactive in FOP [49]. Thus, while senescent cells produce the critical ligands that enhance signaling through the BMP pathway, this same signaling upregulates ID1 and promotes the robust fibroproliferation seen in FOP lesions by the inhibition of p16-mediated proliferative arrest (Figure 1). In this way, ID1, and perhaps other ID genes, may facilitate FOP early lesion formation and the expansion of osteochondral progenitor cells. It is perhaps paradoxical that senescent cells first accumulate after muscle injury to promote BMP pathway signaling, and then BMP pathway signaling prevents p16/INK4A-mediated cell senescence to propagate pre-osseous fibroproliferation.

In FOP, fibroadipogenic progenitor cells (FAPs) from the resident connective tissue stem cell niche are activated by inflammatory signals and they appear to be the major stem cell that amplifies BMP pathway signaling through downstream Smad 1/5 transduction [46]. Although FAPs have been implicated in HO formation in FOP, it is unclear if they become senescent upon tissue injury, are one of the key cell types responsive to SASP reprogramming, or both. Current evidence suggests that both myocytes and muscle satellite cells undergo cellular senescence in response to injury [30,32]. Therefore, the receptive cell required in Urist’s paradigm of HO formation may also be one of the several cell types that contribute to the overall conducive microenvironment in the milieu of senescent cell accumulation and responsiveness to autocrine and paracrine SASP factors.

Elevated BMP signaling mediates a robust fibroproliferative response, perhaps further promulgated by SASP-related platelet-derived growth factor (PDGF) [51,52,53,54] and matrix metalloproteinases (MMPs) [55,56,57,58] (Figure 1). In a tissue-engineered model of HO, PDGF-BB had an osteoinductive effect, as measured by osteoblast differentiation and mineralization [54]. MMPs and, in particular, MMP-9 have roles in endochondral bone formation and may regulate HO formation [55,56,57,58]. Supporting preclinical and clinical evidence strongly indicates that the inhibition of MMP-9 by genetic, biologic, or pharmacologic means abrogates HO formation [13]. In the post-nascent and final stages, the orchestration of lesion formation in a conducive microenvironment and the maturation of FOP lesions result from reprogrammed stem cell-derived osteochondral progenitors (FAPs) that give rise to endochondral bone (Figure 1).

Central to the coordination of fate switching from muscle regeneration to endochondral HO is the association between the dysregulated BMP signaling pathway and inflammatory mediators that either promote or are promoted by senescent cell burden and the SASP. Consistent clinical and experimental observations strongly implicate the innate immune system in the inflammatory exacerbations that are commonly induced in the context of aberrant BMP pathway signaling in FOP. Using connective tissue progenitor cells from patients with FOP and unaffected individuals, we previously evaluated Toll-like receptor (TLR) activation and BMP pathway signaling and found that proinflammatory stimuli upregulate TLR expression, particularly with respect to TLR3/TLR4 [39]. This upregulation amplifies BMP pathway signaling in both a ligand-dependent and independent manner. The integration of TLR and BMP pathway signaling occurs through the Evolutionarily Conserved Signaling Intermediate in the Toll Pathway (ECSIT), suggesting that, in response to injury, FAPs and other cell types may respond to inflammatory mediators directly and perhaps through the acquisition of a senescence-like phenotype. In fact, evidence in support of this hypothesis indicates that TLRs control the SASP in vitro and in vivo [59].

Components of the SASP can influence age-related and injury-induced microenvironmental changes within tissues, both locally and systemically, causing dysfunction. The Janus kinase/signal transducer and activator of transcription (JAK/STAT) signaling pathway is overactive in senescent cells, and its inhibition suppresses and partially normalizes the SASP [60]. The JAK-STAT pathway plays a very important role in the early inflammatory events associated with cell senescence, and the prolonged activation of this pathway results in intense inflammation and fibrosis, potentially contributing to two prominent histological stages in FOP lesion formation via cytokine responses, especially those involved in IL-6 family-related signal transduction [61].

One can hypothesize that the JAK-STAT pathway is activated in FAPs in response to the cytokines released by the SASP after muscle injury and may mediate not only early lesion formation but also tissue reprogramming via IL-6. The JAK-STAT pathway has been incompletely explored in FAPs, particularly with respect to FOP lesion formation. In the normal physiological response to muscle injury, muscle stem cells are primed toward regeneration and repair in an inflammatory microenvironment. In a model of muscle denervation, activated FAPs accumulate, exhibit elevated JAK-STAT pathway signal transduction, and secrete increased amounts of IL-6, resulting in the atrophy of muscle and replacement with fibrotic tissue [62]. Interestingly, FAPs with overactivation of JAK-STAT signaling were identified in a murine model of spinal cord injury, a traumatic event well-known to cause the acquired formation of HO.

The conducive microenvironment that promulgates the initiation of HO lesion formation is a complex interplay among early physiological signals generated by soft tissue trauma (i.e., DAMPS/PAMPS, inflammation, hypoxia) and cellular responses to injury (the accumulation of senescent cells, SASP-mediated inflammation and intensified BMP signaling). The role of hypoxia in the nascent HO lesion, however, is paradoxical.

Although hypoxic tissue modulates cell senescence, limiting its suppression of proliferation and production of SASP factors, it also amplifies the level and duration of BMP signaling and increases the self-renewal and pluripotency of progenitor cells [29,63,64]. This suggests that transient hypoxia primes connective tissue stem cells for reprogramming by the SASP factors produced by senescent cells that begin to accumulate as hypoxia wanes. It is also possible that hypoxia and other early injury-generated signals have their own temporal and spatial cellular targets within the forming lesion with distinct but parallel pathways that merge in orchestrated tissue reprogramming from a myogenic to a chondrogenic fate. (For a thorough review of hypoxia in HO lesion formation, see Wang et al. [65].) This complexity makes it clear that there are multiple key pathways amplified in the early lesion that may be finely targeted, including the senescence phenotype.

## 5. Potential Use of Senotherapeutic Drugs in Disorders of Heterotopic Ossification

Since cellular senescence may have multiple contributions to HO formation in FOP and NHHO, pharmacological interventions that target senescent cells and/or the SASP may be therapeutic candidates [34,66,67,68,69] (Table 1). Drugs that selectively clear senescent cells (i.e., senolytics) were initially identified as inhibitors of pro-survival networks in senescent cells [70]. Chemical agents which normalize or reduce the SASP (i.e., senomodulators or senomorphics), including those that inhibit the JAK/STAT pathway and regulate cytokine production, decrease inflammation in physiologically aged mice (both systemically and in adipose tissue) [35]. Various so-called senotherapeutic (encompassing and referring to both senolytics and senomodulators/ senomorphics) agents have been described and are effective in mitigating or postponing multiple age-related conditions in pre-clinical models. Many of them are currently being studied in clinical trials [34,68,71].

Senotherapeutic compounds fall into several drug classes (Table 1) and, to date, largely represent repurposed drugs. While anti-apoptotic and JAK-STAT/NF-κB pathways are generally targeted by senolytics and senomodulators/senomorphics, respectively, each drug class has off-target effects which make it difficult to identify *a priori* whether or not a given senotherapeutic will be have the potential to mitigate HO formation.

We recently demonstrated that injury-induced cellular senescence and the SASP contribute to FOP lesion formation in mouse models and that tissue reprogramming in FOP is mediated by the accumulation of senescent cells in younger animals, altering myogenic cell fate toward a chondrogenic cell fate [30]. Importantly, the pharmacological clearance of senescent cells abrogates tissue reprogramming and subsequent HO formation [30]. This proof-of-principle evidence suggests that senotherapeutic drugs may hold promise as viable interventional strategies in FOP. At least in the case of FOP, where there is substantial mast cell infiltration of the early lesion [25], some senolytic agents such as dasatinib and quercetin also offer the advantage of being mast cell stabilizers [72,73,74,75]. Senomodulators, such as ruxolitinib and metformin, mitigate the formation of HO in preclinical models [76,77], suggesting that at least some of the therapeutic effects of targeting cellular senescence in HO are related to reducing SASP-mediated factors such as inflammatory cytokines (e.g., IL-6) and MMPs (e.g., MMP-9) [35,78]. Since the JAK-STAT pathway exerts control over the SASP and is also activated in connective tissue progenitors [61,62], JAK-STAT inhibitors may mitigate not only early events in lesion formation but also tissue reprogramming.

Preliminary evidence suggests that not all senolytic agents may be effective in mitigating HO formation. One of us (H. Wang, personal communication) has shown that while dasatinib and quercetin can reduce HO in preclinical FOP models, both singly and in combination [30], the senolytic fisetin has no impact on HO formation. The most likely explanation for this discrepancy lies in the senescent cell anti-apoptotic pathways (SCAPs) utilized by the different subsets of senescent cells and the senolytic agents that specifically target those pathways. For example, dasatinib predominantly targets two unrelated SCAP pathways defined by (1) MDM2, p53, p21, and serpin (PAI-1&2) and (2) ephrins, dependence receptors, and tyrosine kinases, while fisetin targets other pathways defined by (1) PI3Kd, AKT, and protective reactive oxygen species and (2) HIF-1α [34,67,68]. In this way, senolytic agents may also be used as tools to dissect relevant pathways that contribute to early lesion formation in HO.

Activin A is expressed in response to injury in both FOP and NHHO models, but by different types of cells. Although wild type ACVR1 does not transduce signal when engaged by activin A, activin A is nevertheless expressed in NHHO lesions (i.e., traumatic HO). Anti-activin A does not abrogate traumatic HO, whereas antibodies that neutralize ACVR1 or ALK3-Fc (which blocks osteogenic BMPs) partly mitigate the formation of ectopic bone [79]. Nevertheless, since causes of NHHO are largely trauma-induced and senescent cell accumulation occurs in the setting of soft-tissue injuries, senotherapeutics may also be an effective interventional strategy for NHHO, as demonstrated by recent studies [76,77,80]. The rationale for the use of senotherapeutics in NHHO is thus based on shared pathways that are activated in the early response to injury. Since current preclinical evidence indicates that these compounds are effective in the animal models of both FOP and acquired HO, targeting signal transduction through ACVR1, though through different ligands, appears to be mechanistically relevant. In the preclinical models of FOP and NHHO, older animals produce less HO compared to younger animals ([81]; authors’ experience) and this is somewhat conflicting given the presumption that a greater number of senescence cells would be present in older tissues. Possible explanations are that the inflammatory trigger(s) or inductive signal(s) do not reach their respective thresholds to achieve robust HO, at least in older mice.

## 6. Challenges in the Clinical Use of Senotherapeutic Agents for Disorders of Heterotopic Ossification

The weight of evidence for the administration of senotherapeutic agents to mitigate HO favors their use in the setting of potential injury-induced lesion formation. In the case of FOP, this would seem to be most amendable for episodic prophylaxis after trauma or the treatment of trauma-induced flare-ups as soon as they are clinically apparent. However, previous experience with episodic treatment in FOP suggests that it is very difficult to clinically detect early lesions during a window when the likelihood of consistently resolving a developing lesion is high [82]. In the case of acquired HO, when the precipitating event can likely be identified (e.g., trauma) or anticipated (e.g., orthopedic procedure) and is unlikely to be progressive, episodic treatment or prophylaxis with senotherapeutics seems plausible. In both clinical scenarios, further research is needed to enable first-in-human pilot studies.

Since the clearance of senescent cells with senolytic agents appears to occur rapidly in preclinical models (on the order of 1–3 days) [31], episodic and limited treatment may be possible under certain circumstances when the precipitating events are clear. This also minimizes the chance for adverse events associated with longer-term or chronic use. However, given the progressive nature of FOP, episodic treatment with senolytic agents may only be useful with the concomitant treatment of a chronic therapy, whether FOP-specific [83] or with a senomodulator.

## 7. Summary and Future Directions

Genetic and acquired conditions of endochondral heterotopic ossification share similar activating events, early responses to injury, and histological stages leading to mature bony lesions. The accumulation of senescent cells occurs as a result of soft tissue injury, is present in early lesion formation, and may mediate the inflammatory triggers and inductive signals required for tissue reprogramming to a chondrogenic fate via receptive progenitor cells. The SASP may orchestrate a conducive microenvironment where complex interactions among enhanced BMP pathway signaling, fibroproliferation, and reprogramming resolve to replace soft tissue with bone. Cellular senescence may also contribute to aspects of accelerated aging in FOP, independent of HO formation, that may, at least in part, explain disease progression in the absence of obvious inflammatory exacerbations.

Unanswered questions remain in terms of the complete repertoire of cells that become senescent in early lesions; specifically, regarding whether connective tissue progenitor cells such as FAPs become senescent or respond to the SASP of other (local or neighboring) senescent cells, and if reprogramming factors are exclusively derived from cells made senescent after tissue injury. It is also unclear if signal transduction from ACVR1, through different ligands, can completely explain lesion formation in both FOP and acquired HO and what the mitigating factors that determine initiation and completion of the endochondral process in each type are. For example, the inhibition of MMP-9, a SASP factor, abrogates HO formation in preclinical models and in a resilient patient [13]. In the context of cellular senescence as a key regulator of HO progression, one can also hypothesize if the amount of senescent cell burden serves to modulate the threshold required to propagate lesion formation after injury. Finally, the effects of natural aging in patients with FOP and in older individuals who will develop acquired HO, where tissue-specific senescent cell accumulation is expected to increase, are unknown.

The availability of senotherapeutic agents offers the possibility for new interventions in FOP and in acquired forms of HO. The type (senolytics and senomodulators/senomorphics) and specificity of these compounds (targeting different anti-apoptotic JAK-STAT/NF-κB pathways) can serve as both complementary interventions and tools to better understand the lesional cell types that become senescent. As the first generation of these drugs are repurposed agents, their known off-target and safety profile are advantages to exploit as they are translated into the clinic. In some cases, their off-target effects are also appropriately “on-target” as is the case with dasatinib, a senolytic and mast cell inhibitor. One can anticipate that continued research on cell senescence in physiologic aging and age-related conditions will offer further insights into mechanisms applicable to HO, as well as expand the arsenal of senotherapeutics expected in the second- and third-generation iterations of senolytics and senomodulators.

## Figures and Tables

**Table 1:** Selected senotherapeutic agents for potential use in heterotopic ossification.

Type	Class	Example(s)	Mechanism(s) of Action	Comments
Senolytics	Kinase inhibitors	Dasatinib (D)	SCAP trageting	Often used in combination (e.g., D + Q); mast cell stabilizer
	HSP-90 inhibitors	Geldanamycin	Same as class	
	p53/p21 pathway modulators	FOXO4-DRI	Same as class	
	Turmeric derivatives	Curcumin and analogs	Downregulation of Nrf2 and NF-κB	
	Cardiac glycosides	Prosciliaridin A, Digoxin, Strophanthidin	Na^+^/K^+^ ATPase inhibition	May be limited by toxicities
	Galactose modified pro-drugs	SSK1, Nav-Gal	SA-β-galactosidase	
	PROTACs	PZ15227	BCL-XL degadation	
	PPARα agonist	Fenofibrate	Same as class	
	Bioflavanoids	Fisetin, Quercetin (Q)	SCAP targeting (PI3K/AKT)	Mast cell stabilizer
	Antibiotics	Azithromycin	Autophagy and glycolysis inhibition	
Senomorphics	NF-κB inhibitors	SR12343	Same as class	
	P38MAPK inhibitors	SB203580	Same as class	
	JAK/STAT inhibitors	Ruxolitnib, tofacitinib	JAK 1/2 inhibitor	Infectious adverse events
	ATM inhibitors	KU-60019	Same as class	
	Statins	Atorvastatin, Pravastatin	HMG-CoA reductase inhibitor	
	Rapalogs	Rapamycin	Regulation of mTOR, Nrf2, NF-κB	
	SIRT1 activator	Resveratrol	Same a s class	
	Biguanides	Metformin	Regulation of mTOR, Nrf2, NF-κB	Other pathways may also be involved
	Bioflavanoids	Apigenin	IRAK/NF-κB	
		Quercetin	Proteosome activator	

SCAP, Senescence-associated apoptotic pathway.
